# Abstracts and Gleanings

**Published:** 1878-05-20

**Authors:** 


### *ABSTRACTS AND GLEANINGS.*

### EXCESSIVE VENERY.

Prof. Wood in a clinical lecture said: It is difficult to draw the line between proper and excessive sexual relations. What one man can with impunity stand, would entirely break down another's constitution. Excessive venery, though most common among unmarried, is frequently met with in married life. The question will be often put to you by husbands as to how often they should have connection with their wives. With ordinary men, once a week is sufficient. Where, however; both husband and wife are robust, twice a week is not too often. The best rule to adopt in this matter is that the act is performed in excess when its results, exhaustion, etc., make themselves felt. The normal act should leave no trace behind.

The symptoms of excessive venery are those of general debility. In some cases there may be slight spermatorrhœa. There is weakness about the loins, back and lower limbs. In severe cases there is loss of power in the lower limbs, almost amounting to palsy. Excessive venery is probably always attended with some molecular change in the nerve centres. After prolonged abuse, organic changes, such as myelitis, locomotor ataxia, and chronic sclerosis take place. The case which I have taken as a heading for my lecture is a very good example of the more marked symptoms of excessive venery. Although, as I have said, excessive venery is occasionally the cause of organic changes in the cord, yet I am inclined to believe that not infrequently tt is a result, rather than a cause, of commencing neural disease. Paraplegia following excessive venery is rare.

In some rare instances the brain is affected, with cerebral softening or epilepsy as a result. In epilepsy from this cause the aura is more distinct and travels more slowly than in idiopathic epilepsy. So that if the aura begins in the forefinger, for instance; there is time enough usually to grasp the wrist firmly and to prevent the seizure.

### TREATMENT OF EXCESSIVE VENERY.

Of course the practice must be stopped. In some cases it becomes necessary to insist that husband and wife sleep in separate beds. All coition must be absolutely forbidden until perfect virility be regained. As regards hygiene, nourishing food, warm clothing and plenty of sunlight and exercise are indispensables. Where the emissions are numerous, the same hygienic measures as in cases of masturbation must be employed. Medicines may be given, first to cure the disease, and second to aid in the moral effort at continency. For the first purpose iron and the bitter tonics are indicated, while to subdue all excitement and local irritability the bromides may be given up to the point of producing bromism. A specific remedy is phosphorus. It may be administered alone or with ergot. The ergot is very plainly indicated where there is numbness or prickling of the limbs. The action of phosphorus in some cases is really wonderful. Do not, however, give phosphoric acid and think that you are giving phosphorus. Where the disease has gone on to organic spinal disease you must treat the symptoms on general principles.

### IMPOTENCE.

I want, in conclusion, to say a word to you on this subject. There are two kinds of impotence: 1. That connected with excessive irritability of the organ, and 2. where there is loss of power without irritability. We usually meet with the first form in young men who have been in the habit of masturbating before they were married. In these instances emission occurs before, or just after, intromission. The proper treatment in such cases is continued doses of the bromides. The patient must be also warned against marital excesses. In the treatment of the second form the following is a good remedy :

℞.—Tinct. canthar., gtt.vj.
Tinct. ferri chloridi, gtt.xv-xx.
M. Sig.—Thrice daily, in water.

In impotence with spermatorrhœa the tincture of cantharides acts like a specific. The cantharides should not be given where debility is absent. If further treatment be then required, cold bathing strychnia and electricity may be employed.

---

REMARKS ON OBSTETRICAL OPERATIONS.

Prof. Walker, in a paper read before the Medical Society at Evansville, says :

Version has generally been resorted to in vertex cases, when the head was still above the brim and immediate delivery became necessary, from the belief that it was safer for the woman than the forceps, that it could be practiced when an attempt at forceps delivery might fail, and that even to the child the danger from delivery by turning would be no greater than by the forceps when applied above the strait; and finally, because the operation in many instances would be more expeditious. Even after the head has descended into the upper strait, and is still easily pushed up above the brim, turning has sometimes been preferred, especially where the child was dead. Turning, as compared to cephalotomy, has been regarded as safer for the mother, the cephalotomy being a dernier resort, and to be used only when turning is impracticable. In bringing down the feet, securing a single foot has been deemed sufficient, if for no other reason to save the valuable time necessarily consumed in searching for the second foot, and also from the belief that by leaving the second limb folded against the abdomen, some protection is afforded the funis against undue pressure. Ample experience has convinced the writer that the delivery can be effected as well by securing one foot as two.

In placenta prævia cases, the indication has been supposed to be two-fold : 1, to arrest hemorrhage, that threatens the woman's life ; and 2, to save the child's life. To secure these advantages, delivery by turning as soon as it can be done, without using too much violence in the introduction of the hand, is the chief, if not the only resource. Therefore, at the earliest practicable moment, the hand is to be passed through the crevix into the cavity of the uterus, detaching the placenta from its connection as little as possible, and when the membranes are reached, the fingers are to be pushed through, search made for the child's feet, or rather foot, which being brought into the vagina, the child is to be cautiously taken away. The operation need not be delayed on account of the absence of labor pains, nor of the immature state of the gestation. To wait in these cases for the natural process of labor, would result too frequently in the double loss of the mother and child.

As before stated, the forceps have not often been appled above the brim, this mode of using them, indeed, appearing more intended to show what may be done than for any special advantage or indication. After the head has passed the superior strait, as a general rule, the forceps will be the best and safest remedy ; and, should the head have been pushed into the excavation by strong pains, and yet its expulsion not promised by the efforts of nature, the forceps would be indicated, and turning should not be thought of. The writer has never applied the instrument to any other part of the child than the head ; the barbarous practice of applying them to the pelvis, should the child be delivered alive, might so crush the bones as to endanger permanent danger to the pelvis. The forceps, then, being applied to the head only, should be as nearly as practicable adjusted to its sides. The writer has never met with a case in which application over the face and occupit was necessary; when convenient, the blades have been adjusted to the sides of the woman's pelvis, but this has been deemed less important than to permit much obliquity in their application, as regards the child's head. Indeed, in oblique positions of the head, which constitute more than ninety out of one hundred of the cases, it is impossible to apply the instrument squarely to both pelvis and head. When practicable, the forceps have been removed before the escape of the head from the vulva, to avoid laceration; but this practice is more easily recommended than followed, inasmuch as the moment the head has engaged fairly in the outlet, it will frequently escape through the vulva, even before the operator finds himself able to remove the instrument. Doubtless lacerations are more frequent than they otherwise would be, in consequence of this failure, but in most cases requiring the use of the forceps lacerations would be liable to follow a natural delivery.

Cephalotomy has been recommended by the writer, only in those cases in which neither turning nor forceps could be made available. It is not regarded as a safe operation for the woman, and when a fatal result is avoided she is necessarily exposed to more or less danger of laceration about the crevix and vagina, so that after the bulk of the child's head has been reduced by the evacuation of the brain, the delivery may be completed more safely and more expeditiously by podalic version than by tractions with sharp and powerful instruments. In addition to this, we may urge the risk of bruising and tearing the soft parts by drawing away fragments of the fœtal skull, that are almost certain to become loosened during the transit of the head.

When the cephalotribe is required in extreme contraction of the straits, a resort to turning will generally be inadmissible, from the difficulty or impossibility of drawing a full-sized child through the pelvic canal. When, indeed, it is found necessary to use this instrument to crush the head, its power will generally be required also to reduce the other bulky parts of the child.

---

HYDROBROMIC ACID IN CEREBRO-SPINAL MENINGITIS.

The many excellent qualities of this acid which render it so useful a member of our therapeutical armamentarium, especially in fevers accompanied with considerable disturbance, make it incumbent upon us to refer to it briefly in this issue. There has been considerably desulotory writing in the journals concerning it during the past few months, all pointing to its excellent qualities as a cerebral sedative, and tranquilizer of the nervous system. It possesses all the beneficial action of the bromide of potassium, without the relaxing effects of the potash, and does not superinduce boils. It does not stimulate as does bromide of ammonium, and may be readily combined with quinine, to produce the hydrobromate of quinine, a most valuable tonic to the nervous system in low forms of fever, etc.

To Dr. Fothergill of London, Eng., belongs the credit of fiast having separated this acid for use, since which time it has excited considerable interest in medical circles. He gives the following formula for its production in quantities of two quarts: dissolve oz.xj of bromide potassium in four pints of water, then add oz.xiij of tartaric acid. A precipitate of bitartrate of potash falls down as a sediment, and the hydrobromic acid remains in a clear, bright, almost colorless fluid, possessing an acid taste and the ordinary acid properties, and is possessed of the peculiar therapeutical properties of bromide of potassium, as distinguished from those of any other salt of potash. The dose of this acid, thus prepared, is from half a drachm to a drachm. The smaller dose is usually that employed, except in severe cases. It is the form of bromine best suited for use in medicine. It is commending itself in the South as a remedy in fever, combined with large anti-pyretic doses of quinine. In the

*Peninsular Journal of Medicine,* Dr. Wade recommends its use in the treatment of fevers, and says "it would seem the acid *par excellence* when there is much cerebral excitement, in pyretic affections."

In cerebro-spinal meninigitis, we have a specific contagious virus of a typhous nature attacking with especial virulence the great nerve centres. To treat this successfully requires the highest skill and the greatest promptitude and aptitude in the selection of remedies. Briefly, we may here summarize the most recent conclusions of the ablest men in the professions as to its treatment, as in this we may best shew the place and power of this acid, as an agent in the treatment of this formidable affection.

First: the hyperæmia of the brain and spinal cord should be relieved by the prompt and repeated application of leeches, until the pulse has fallen to below 100 or within a point at which it ceases to be alarming. Second; hot applications (not cold) are to be applied to the head and spine, with mustard pediluvia. Third; the bowells should be unloaded with an active cathartic. Fourth; to relieve the hyperpyrexia (the temperature being sometimes as high as 104° or even 106°) sedative doses of quinine (say 2 to 5 or even 10 grs.) dr.j doses of the hydrobromic acid should be administered frequently, and continued until the petechial spots have disappeared from the skin, in doses of course, commensurate only with the hyperpyrexia or excess of heat.

Some prefer a solution of quinine in hydrobromic acid which may be administered in doses of from ½ to 1 drachm of the acid, and 1 to 2 grs. of quinine hourly. The surface of the body should be regularly sponged as in other fevers. It is claimed that this mode of treatment will save over 75 per cent. of such cases, and prevent the distressing sequelæ which sometimes follow, showing defective nerve power. The leeching is indispensable to relieve the violent head symptons at the outset, and the antipyretic properties of the quinine are needful; but without the acid neither of these remedies would prove of more than temporary benefit.

The use of calomel has been much lauded by some, but is rapidly falling into disuse as unnecessary. Opium in moderate doses is of great service in the later stages of the disease and assists materially in promoting convalesence.—*Canada Lancet.*

---

### CHLORIDE OF ZINC IN THE TREATMENT OF ULCERS OF THE LEG.

The numerous methods which have been tried for the treatment of ulcera cruris, show plainly how great is the number of those who suffer from this affection, and how these ulcers defy all remedies.

Reverdin conceived the idea of transplantation of the skin, which, however, has not proved valuable in these cases; Fidders would hasten the cure by sprinkling pulverized skin on the granulations, and Macleod obtained only negative results by moistening the ulcers with the lymph from a vesicle produced by vesication. The repeated incision of the ulcer and the cutting out of its base, led likewise to success.

Therefore Dr. Gurbski in Plock has for the past three years applied a salve made with equal parts of chloride of zinc and meal, with the best results. From the list of caustics he has chosen the chloride of zinc, because its action is limited, and because a layer of paste can be applied just as thick as the part which is to be destroyed. The ciatrices are soft, pliable, and not disposed to break down where there is dilatation of the veins, or to form new ulcers.—Gurbski gives only two cases, since all had a similiar course.

(1) A maiden 23 years old showed at the time of her arrival, April 27th, an ulcer of five years standing, on the under and inner side of the right leg, elliptical in shape, 8 cm. long, and 5 cm. broad. The surface of the sore is smooth, grayish red, surrounded by thick hard borders. Besides this, there were upon the outer side thirteen ulcers, varying in size from a lentil to a half dollar. A paste 5 mm. thick was placed upon one half of the larger sore, and two grammes of chloral given internally. In order to hasten the removal of the scab, a cataplasm was applied. On the 30th of April, it fell off, and then the other half was covered with zinc paste. On the 7th of May, this scab also fell off.

On the 11th of May, the first half, and on the 21st, the entire ulcer had cicatrized. On the 14th of May the paste was applied to the thirteen smaller sores, so that on June 14th, the patient was discharged, entirely cured. (2) A maiden 20 years old, had on the 15th of April, six ulcers, of two years standing; five were the size of a dollar, and one, of a quarter. Four of them were immediately covered with paste; after five days the scabs fell off, whereupon the two remaining ulcers were put under treatment. On May 28th, all the ulcers were cicatrized.—*Clinic.*

---

### RETAINED PLACENTA IN ABORTION.

Dr. May (in *N. Y. Med. Rec.*), remarks:

Taking for granted that the fœtus has been expelled, and we have to deal with a retained placenta attended with hemorrhage more or less severe, the tampon —made of strips of "old muslin," eight or ten inches square—is resorted to, the vagina being packed from its vault to the vulvæ, and secured by a T-bandage. This soon absorbs the blood in the vagina, and, if well applied, is an absolute protection against further dangerous bleeding. The further loss of a few ounces of blood in many of these cases might prove dangerous, if not fatal.

In about twelve hours the tampon is removed, and usually the placenta will be found attached to the first cloths inserted. If not, the vagina is syringed with carbolized warm water and the tampon is removed, using, of course, fresh cloths lubricated with olive oil or castile soap and warm water. After remaining twelve hours longer the cloths are removed, and I have always found the placenta in the upper vagina ready to be brought away with the tampon. I never have found it necessary to repeat the tampon the third time. Its presence softens the os, excites moderate, almost unconscious, uterine contraction, and enables the physician to leave his patient for other duties, confident in the perfect immunity from danger in which he leaves the valuable life entrusted to him.

I may be better understood by reporting in this connection the following typical case which has just occurred in my practice. I treat a goodly number in the same way every year and with equal success.

Mrs. A. B., aged thirty-two, American, in good circumstances, married eight years, has borne four children at full term, and, with this, has suffered four miscarriages. Was called to this lady at 10 A.M., March 24th, ultimo. She informed me that she thought she was nearly four months on in pregnancy. For a week she had daily noticed a slight vaginal discharge of blood. Her children had the whooping-cough, and she had been broken of her rest a great deal in caring for them. She had that morning been about as well as usual attending to household cares, and while standing at a table she felt a sudden escape of blood from the vagina, and went immediately to bed. She fainted quite away before my arrival. I found her pale and prostrate, with feeble, rapid pulse. A large amount of blood saturated the bed. Included in these clots was a small fœtus about three inches in length. The vagina was filled with clots, and blood escaped freely from the uterus. This slight examination induced extreme syncope. I at once "plugged" the vagina, as above detailed. Squibb's f. ext. of ergot was given, which excited vomiting; but this effort acted favorably by forcing blood to the brain. I remained with my patient three hours, using restoratives vigorously, and striving to relieve the deadly nausea and depression. Our efforts were successful, and a good degree of reaction secured. At my evening visit I found no hemorrhage had occurred, and the patient so comfortable that it was decided to leave the tampon *in situ* until morning.

At 6:30 A.M., on the 25th, the cloths were, one after another, removed, saturated with dark blood, and the entire placenta was found in the vagina attached to the upper cloths. The uterus was found well contracted and closed. My patient is making a rapid recovery.

Had I at my first visit persisted in getting away the placenta "at all hazards," by the method suggested by Prof. Penrose, I feel almost sure that a valuable mother and lovely woman would have been lost to her young children and numerous friends.

---

TREPHINING FOR COMPRESSION.

Dr. Rodman (in *Med. Record*), reports the following case:

Berry Henderson, colored, age seventeen, received at 11 P.M., Sept. 24, 1877, a blow on the left side of the head two inches above and slightly in front of the ear, from a comparatively flat smooth stone. He was knocked down, but was able, with assistance, to walk home, a distance of several hundred yards. At 12 midnight Dr. Phythian saw him. He was then beginning to recover from concussion of the brain. There was neither scalp wound nor fracture of the skull; a slight contusion alone indicated the seat of injury. As there were no symptoms of compression, and those of concussion improving, the doctor left without doing anything for him. The next morning, at 9 o'clock, he was found by Dr. P. to have decided convulsive movements of the right side and serious symptoms of compression. These had commenced about 1 o'clock the night before, only a few minutes after the doctor had left. Purgatives, cold to the head, and large doses of the bromide of potassium were ordered. The two latter were kept up until late in the afternoon. The symptoms continuing to grow worse it was determined to cut down upon the skull and search for a fracture. The scalp being incised and reflected, no fracture could be found. In this dilemma the bromide and cold water were continued with the understanding that if the smptoms were not materially relieved by morning trephining should be resorted to. Morning came and with it an increase in the severity of symptoms. Upon consultation trepining was unanimously advised. The instrument was applied to the skull immediately under the contused scalp, the necessary incision having been made the day before. Upon lifting out the disc of bone, the clot was plainly to be seen between the dura mater (which was uninjured), and the skull. There being some difficulty in removing the clot through the opening already made, another disc was taken out. The clot extended in every direction beyond the edges of tne opening. Only about an ounce of it could be removed, but we hoped that this, together with the relief of pressure from the absence of bone, would be sufficient. While the boy's condition was not improved as much as we desired, still there was sufficient abatement of the symptoms to justify the operation and lead us to expect recovery. In a few days he was out of all danger, and in due time fully recovered. He is now engaged in farm work near Frankfort. He suffers no inconvenience from any cause. That he would have died without operative interference is almost as certain as that he recovered with it.

I conclude that a surgeon is perfectly justified in trephining for the relief of compression of the brain from extravasation of blood (with or without accompanying fracture of the skull), provided, 1. That the clot is meningeal; for the necessary diagnostic differences refer to Erichsen, loc. cit. 2. That the clot can, with *some degree* of certainty, be localized. For instance, if a man is struck with a missile, injuring more from its

*velocity* than *weight*, and extravasation is the result, I think it would be safe to trephine over the injured spot. If a man were to fall from cars in motion, from a horse, out of a window, or over a stairs, where the weight of his body produces the injury to the head—in other words, if the extravasation is possibly the result of counter stroke, and upon examination no decidedly localized point of contact can be found, the propriety of trephining becomes very doubtful. That cases of extravasation uncomplicated by fracture in which an operation is admissible are *very rare* cannot be denied. But that all such cases should be treated on the medicinal or expectant plan I cannot believe.

———

ARSENIC IN THE TREATMENT OF MALIGNANT TUMORS.

Of all agents recommended in the treatment of cancer, I still believe arsenic to be the most efficient, having administered it internally and applied it locally in desperate cases, and sometimes not without good results. Prof. C. M. Langenbeck related the case of a patient afflicted with a cancer of the uterus of such dimensions as to preclude the possibility of an operation. Arsenic had been recommended the patient who, in the hopes of terminating her existence, took it in rapidly increasing doses; in this she failed, and after a few months L. was enabled to confirm a perfect cure of the disease. I frequently administered the remedy, at first in cases where operations were not practicable, and later after successful operation to prevent relapse, and I believe that occasionally these did not appear in cases where I dreaded their early return. The views entertained to-day concerning the nature of cancer indorse the administration of arsenic in a certain measure on a rational theory. That arsenic taken internally has a certain curative influence on various diseases of the epidermis no one can deny. If we consider cancer as an abnormal proliferation of epithelium principally it is very natural to resort to arsenic for its cure. The researches of Gaethjens who, after liberal administration of arsenic, noticed an increase in the excretion of nitrogenous substances consequent on disintegration of albumenoids, speak for its use against malignant tumors. We must not expect, however, to accomplish much with the minute doses recommended in the text-books. If in hopeless cases visible results are to be obtained we must allow the arsenic to combat the disease for life and death. In this way administered I have repeatedly witnessed remarkable effects. A patient presented herself at my clinic with a cancer of the upper jaw with such dimensions that I dared not operate. I ordered arsenic in rapidly increasing doses. A few months later she presented herself again with large cicatrices in the face reporting that a few weeks previous a large portion of the tumor had sloughed away, a rapid closure of the wound following. That the diagnosis was correct was confirmed by the fact that a year later a recurrent cancer supervened in the cicatrix, and extending rapidly soon caused the death of the patient. The results which Billroth and others have obtained from the internal administration and external application of arsenic in malignant multiple lymphoma prove that arsenic is beneficial not only in tumors of epithelial origin; in a number of cases of lympho-sarcoma I have been enabled to establish its efficiency.—Esmarch—*Langenbeck's Archiv.*

———

CHLORAL BY ENEMA.—Certain experiments with chloral go to show it can be taken up by the absorbents of the lower bowel with nearly the same energy as by the stomach. In a case of pueperal convulsions, a solution of chloral and brom. potas. was injected per rectum, with the result of allaying spasm promptly and decidedly. Thirty grains in two or three ounces of water will commonly suffice for a first injection.—*Proceed. County Kings.*

### KOUMISS, GLYCERINE AND CREOSOTE IN CONSUMPTION.

The three agents above named have attracted recent attention in the treatment of consumption. *Koumiss* is said to differ from milk only by the addition of the three products developed by fermentation, to-wit: alcohol, carbonic acid and lactic acid. It is claimed that it improves the appetite, relieves nausea, cough, and night sweats, and increases the weight of the body.

*Glycerine* has been recommended in doses of 15 to 30 grammas per day as a substitute for cod liver oil, and has been found to build up the system and diminish the excessive sweats and waste of the organism.

*Creosote.* In a paper translated from the French by R. B. Davy, it is stated that "Creosote differs from koumiss and glycerine in not appearing to affect the healthy organism very perceptibly, while the two others are characterized by the difference in the amount of urea and increase in the weight of the body. Bouchard satisfied himself of this in a healthy adult weighing 65 killogrammes. The condition of the principal functions and amount of urine were noted for 34 consecutive days. During the first 27 days the average amount of products elminated was determined and during the last 7 was administered morning and evening, creosote 20 centigrammes, (3 grs.) in an alcoholic solution, one part to the thousand. Thus Bouchard saw that the subject had not changed in weight, but he respired a little more slowly and the quantity of uric acid was diminished to about one-third.

"The essential condition to the good administration of creosote is to give it well diluted and perfectly dissolved; for it must not be forgotten that it is an energetic caustic." W.

---

### POLYURIA SUCCESSFULLY TREATED BY ERGOT OF RYE.

The polyuria in a case reported by Dr. Rendy (*France Medicale* Feb. 27, 1878) was accompanied by supraorbital neuralgia, vertigo with the loss of consciousness, excessive thirst and hunger, with emaciation and loss of strength, although the patient consumed a considerable quantity of food. The urine contained no trace of sugar; the quantity was about ten quarts a day. The urea eliminated by this means in the twenty-four hours amounted to from about 1,250 to 1,400 grains. Before having recourse to ergot of rye, tincture of valerian was first tried for this patient, in the dose first of fifteen minims, and soon afterwards of half a drachm. Under the influence of this treatment, the urine diminished by nearly a quart. Sulphate of atropine, in the dose of one *milligramme* (0.15 grains) at first, then two, daily, produced a similiar improvement; but no advantage was found in persevering in this course, since the appetite diminished with the valerian, and the thirst increased with atropine. Ergot of rye was then tried. The success with this agent was remarkable. In eight days, the urine fell to 1,600 *grammes* and the urea to 15 *grammes* in the twenty-four hours; the emaciation was stopped; the strength returned; whilst the thirst and the excessive desire of food also disappeared. Dr. A. Costa (*N. Y. Hospital Gazette*, Feb. 15) reports also a case of diabetes with the excretion of ten pints of urine daily, with sugar or albumen, marked by great emaciation; and states that he treated the patient with fluid extract of ergot, which treatment had been followed by striking success; *i. e.*, complete cure in two cases in private practice. Dr. A. Costa put the patient upon an initial dose of half a drachm of the fluid extract daily, the dose to be increased gradually, first to one drachm, and then to two drachms. There was at once apparent a great reduction in the quantity of urine passed daily. From ten pints, it fell to six pints daily; then to three, where it remained. Even before reaching the present limit, he ordered the dose to be gradually reduced, first to one drachm, and then to half a drachm. Then it was stopped altogether, and mint-water substituted in its place. For the past two weeks, he had had no ergot, and might be considered permanently cured. The amount of urine daily passed varied between two and three pints.—*Brit. Med. Journ.*, April 13, 1878.

### ABSORPTION OF TINCTURE OF IODINE BY THE SKIN.

Dr. L. Menager has experimented upon children with a solution of equal parts of tincture of iodine and glycerin, rubbed into the skin, and has arrived at the following conclusions:

1. Iodine in tincture mixed with glycerin and applied to the external integument is absorbed.
2. This absorbed iodine is invariably found in the secretions and in the urine. (Dr. M. tests for iodine by adding a little starch to the urine in a test-tube and then dropping a few drops of nitroso-nitric acid into it. This give a blue or violet color according to the quantity of starch present.)
3. This application may give rise to certain symptoms, usually a variety of mild temporary albuminuria.
4. Dressings containing tincture of iodine may be employed as a means of introducing this medicine into the system when it cannot be taken by the stomach.
5. It must not be forgotten that when this absorption takes place in patients subject to nervoso-vascular erethism, as in certain cases of phthisis. where these dressings are often practised, they may do more harm than good.—*Med. Times.*

### VIBURNUM PRUNIFOLIUM.

The fluid extract of Viburnum prunifolium is mostly employed as a prophylatic in threatening abortion, and in cases of habitual abortion, in doses of ½-1 teaspoonful four times daily. In dysmenorrhœa, accompanied with pain and great loss of blood, it greatly alleviates the symptoms if administered from a few days before, until a few days after menstruation. In cases of spasmodic or neuralgic dysmenorrhœa it should be combined with sedatives. The fluid extract should be prepared from the bark of the root and the yonng branches. The ordinary dose is 1-8 to 3-75 grammes (½ to 1 drachm) every 2 to 6 hours.—*Gynæcol. Trans. in Ph. Zeit f. Russel.*

### THE LATEST CONCERNING GERMS.

In the Academy of Medicine of Paris they have recently been having a very animated discussion on disarticulation at the hip joint, and on the treatment of wounds in general. Of course fermentation, putridity and germs could not long be the subjects under discussion without eliciting some remarks from M. Pasteur. He arises and astonishes the medical world with the declaration that air kills the germs of septicæmia. For the poisonous principle of pyæmia we must analyze water, and a communication which is forthcoming from him will establish the fact that it is necersary to proscribe it as the most dangerous of poisons for wounds.—*Clinic.*

### BORACIC ACID IN SKIN DISEASES.

Having had his attention drawn to this remedy by a recent publication of Nystroin, Dr. Neumann (*Centralblatt f. Chirurgie*) instituted a series of experiments with it. He sometimes employed it alone, at others in conjunction with cloves, in the form of fluids and of ointments. In pityriasis versicolor, and herpes tonsurans he used a spirituous solution of one part to thirty with the addition of a small quantity of oil of cloves; in all varieties of eczema in the form of salve. He considers this agent a valuable addition to our resources and indicates his intention of further investigating its virtues.—*Clinic.*

THE SENSE OF SMELL may, according to Dr. Dupy, of New Zork, aid in the diagnosis of tubercular meningitis; patients having that disease emit an odor closely resembling wet linen. The odor in tetanus, on the other hand, is that of a wet cloth.—Letter of Dr. P. B. Porter to the *Med. Times*, Nov. 10th.

### A REMEDY FOR THE ERUPTION OF POISON OAK, IVY, AND SUMACH.

Dr. S. A. Brown, U. S. N., More Island, California, believes that he has found a specific for the eruption caused by contact with poison oak, sumach, ivy, hua-hoo, cashew nut, etc. He writes:—"This specific is *bromine.* I have used it with the same unvarying success in at least forty cases. The eruption never extends after the first thorough application, and it promptly begins to diminish. Within twenty-four hours, if the application be persisted in, the patient is entirely cured. There is no pain attending its use, as from that of astringents. Of course, the epidermis peels off as after other treatment.

"I use the bromine dissolved in olive oil, in cosmoline, or in glycerine. The application with glycerine is painful, and, I think, possesses no advantage to compensate for the irritation. The strength of the solution is from ten to twenty drops of bromine to the ounce of oil, used by rubbing gently on the affected part three or four times day, and especially on going to bed at night. You wash off the oil twice a day with castile soap.

"The bromine is so volatile that the solution should be renewed within twenty-four hours of its preparation, as it will get out of a bottle, however well corked. It is best to stand the bottle on its corked end, in the intervals of application.

"I have seen no publication of this treatment, and I, therefore, send you my experience with it, hoping to attract to it some little attention, and do the good which must result from its adoption."—*Med. Record.*

### RAPID EXPULSION OF TAPE-WORM.

Dr. Pauline in a communication to the *Allg. Med. Central-Zeitung.* No 21, 1878, narrates a case in which the action of the anthelmintic was remarkably prompt. One of his convict patients having informed him that he was suffering from tape-worm, he immediately ordered the following: R Flor. Kousso dr.vj, Kameela dr.iv. Half of this powder was taken immediately in water; this was at eight o'clock in the morning. At nine o'clock he took a dose of Carlsban salts, and one hour later, the remaining half of the powder. No nausea or vomiting was produced. About half after eleven o'clock, free evacuation took place and in the stool was found a coil of several tape-worms. Four heads of the tænia solium were delivered in addition to a large number of lengthy segments.—*Clinic.*

### HŒMOPTYSIS.

A writer in the Boston *Medical Journal* says: The sovereign remedy against hæmoptysis is ergotin, which, as is well known, excites the vaso-constrictors. A solution in glycerin (1:10) is better than a solution in water, as after long standing it shows but little sediment and no fungi. After the injection the spot injected becomes very sensitive, with some heat, followed by redness, which disappears in eight or ten hours. If the patient is much excited, or has much cough, the author is accustomed to precede the ergotin injection with one of morphia, or to give them both at once, but in different places. In this way, the patient becoming quiet in mind and body, the ergotin has a better chance to act.

### INSTANTANEOUS CURE OF HYDROCELE.

Dr. Macario, of Nice, contributes some interesting cases treated by electro puncture. In the first case, two needles were plunged into the tumor, one at the base and the other at the apex. On connecting the needles the pain was such that the patient refused to continue treatment. Nevertheless, the next day the liquid had disappeared and had not returned at the end of nine years. In the next case absorption was even more rapid, a tumor the size of two fists, dating from fifteen months, having vanished in the evening after a single sitting of one minute. Dr. M. has also reported to the institute several other cases treated, some by electro-puncture, others by simple induced currents, and it is more than fifteen years since he first recommended this method, which has been followed by several others with considerable success. — *Physician and Pharmacist.*

---

DIARRHŒA IN INFANTS.

Dr. Rene Blanche *Bull. Gen. de Therap.*, 1878, p. 89; from *Jour. de Therap.*) urges that whenever diarrhœa occurs in young infants it should be checked immediately and not allowed to make headway. The medicine he employs is the same in every case, though modified somewhat according to circumstances. In order to prepare for this, diminution of the ordinary diet is directed, and appropriate enemata after each passage, with cataplasms to the abdomen. Then every morning a small teaspoonful of an emulsion made of equal parts ol. ricini and syr. acaciæ is given, and repeated every day for three, four or five days. For infants under six months, scru. j. ol. ricini is enough; from six months to two years, oz. ss. to oz. j. If after a day or two the stools improve, the dose is maintained, but if they are still fetid and glairy, an equal dose may be given in the evening as well as in the morning. When the passages are very frequent, one to three drops of laudanum may be added in the course of twenty-four hours. M. Blanche thinks enemata very important. A large enema of infusion of chamomile may be given at the outset, followed by a smaller one of starch, twenty minutes later.—*Philadelphia Medical Times.*

---

ARSENIC AND ITS PREPARATIONS IN THE TREATMENT OF SKIN DISEASES.

Molinari, in an article on this subject, (*Gaz. Med. Ital.-Lomb.*, March, 1877; *Jour. des Sci. Med.*, 1878, p. 100), concludes as follows: 1. Arsenical preparations should be administered at first in small doses, which are to be increased gradually, their effect being carefully watched. At the first sign of disturbance, as loss of appetite, pain in stomach, dryness of the mouth, swelling of the eyes or nose, or difficulty in urination, the medicine should be suspended, but renewed again when these disappear. 2. A saline purgative, as sulphate of sodium, should be taken before the beginning of the treatment and at its close. 3. Arsenic should not be given after, but before, meals; it is better tolerated under these circumstances, and is more quickly absorbed. Acids should not be taken by the patient, and alcoholic drinks only rarely. The treatment should last from one month to six weeks. 4. External means, as ointments, etc., should be added to the arsenical treatment. 5. In eczematous affections, where the kidneys are involved, some diuretic, as the acetate of potassium, may be added to the arsenic, but not substituted for it.—*X., in Philadelphia Medical Times.*

---

BISMUTH IN THE TREATMENT OF PROLAPSE OF THE RECTUM AND HEMORRHOIDS.

A case is reported in *La France Medicale*, No. 86, p. 682, where a considerable protrusion of the rectum was perfectly cured by means of bismuth powder used locally. The physician introduced every day into the bowel, after replacing it, a small spoonful of bismuth (subnitrate?) in a small amount of starch-water. Cure followed in a week. Good results followed similar treatment in cases of prolapse in children, and in hemorrhoids.

---

FIBROID POLYPUS OF THE VAGINA.

E. Cross, M.D., (St. Louis *Clinical Record*), reports a case of this rare affection that has been mistaken for inverted uterus. The growth was nearly as large as a child's head, and protruded beyond the valva, the pedicle was small, and attached one and a half inches from the ostrical to the vaginal wall. Removed by double ligature and ecrasure with perfect success. Dr. C. shows how barren our literature is on such growths, Dr. J. Marion Sims having only seen one case.

